# An Assessment of Psychological Noise Reduction by Landscape Plants

**DOI:** 10.3390/ijerph8041032

**Published:** 2011-04-07

**Authors:** Fan Yang, Zhi Yi Bao, Zhu Jun Zhu

**Affiliations:** 1 College of Agriculture & Biotechnology, Zhejiang University, 268 Kaixuan Road, Hangzhou, 310029, China; E-Mail: yf19843611@163.com (F.Y.); 2 School of Landscape Architecture, Zhejiang A & F University, North Huancheng Road 88, Linan, Hangzhou, 311300, China; 3 School of Agriculture and Food Science, Zhejiang A & F University, North Huancheng Road 88, Linan, Hangzhou, 311300, China; E-Mail: zhujun.zhu@zjfc.edu.cn (Z.J.Z.)

**Keywords:** green transportation, environmental therapy, electroencephalogram (EEG), psychological noise reduction, urban green space

## Abstract

The emphasis in the term ‘Green Transportation’ is on the word ‘green’. Green transportation focuses on the construction of a slow transport system with a visually pleasing, easy and secure trip environment composed of urban parks, green roadside spaces and some other space that is full of landscape plants. This trip environment encourages residents to make trip choices that reduce fuel consumption and pollution and is one of the most important ways of popularizing green transportation. To study the psychological benefits provided by urban parks and other landscape environments, we combined a subjective approach (a questionnaire) with an objective quantitative approach (emotional tests using an electroencephalogram; EEG). Using a questionnaire survey, we found that 90% of the subjects believed that landscape plants contribute to noise reduction and that 55% overrated the plants’ actual ability to attenuate noise. Two videos (showing a traffic scene and a plant scene) were shown to 40 participants on video glasses. We detected and recorded EEG values with a portable electroencephalograph, and a comparison between the results of the two groups revealed that there was a highly significant asymmetry between the EEG activity of the vegetation scene and traffic scene groups. The results suggest that the emotions aroused by noise and visual stimuli are manifested in the synchronization of beta frequency band and the desynchronization of alpha frequency band, indicating that landscape plants can moderate or buffer the effects of noise. These findings indicate that landscape plants provide excess noise attenuating effects through subjects’ emotional processing, which we term ‘psychological noise reduction’.

## 1. Introduction

The threat of a global energy shortage together with issues of air pollution and noise pollution have made ‘Green Transportation’ an increasingly popular concept. The main focus of green transportation is on the word ‘green’ rather than on the word ‘transportation’. An aim of the green transportation movement is to achieve the goal of sustainable urban transportation, which is defined as “efficient, equitable, secure, eco-friendly, low consumption” transportation [1]. Many studies have found that encouraging bus priority and constructing slow transport systems are the most effective means of establishing green transportation systems. Providing a green, easy and secure trip environment is the foundation of the slow transport system. This paper evaluates roadside green spaces and sidewalks as sample trip environments, presents new ideas for the evaluation of psychological noise reduction of landscape plants, and aims to lay a theoretical foundation for constructing green trip environments. Noise pollution is one of the public hazards considered to be a cause of widespread occupational and community health problems in both developed and developing countries [2]. Many studies have been performed on noise control, and they have focused on issues such as the following: the calculation, simulation and measurement of street sound environments; noise control technology for road traffic noise; acoustical insulation of buildings and noise control regulations [3]. The control measures that have been taken have mainly focused on the physical control of environmental noise. Vegetation has been regarded as a cheaper and more natural material to reduce outdoor noise pollution in comparison to concrete, metal, plastic and other such man-made materials. Although techniques for noise reduction continue to improve, there are increasing complaints regarding noise interference. The ultimate goal of noise control is to promote relaxation, satisfaction and well-being in urban residents.

Previous studies have indicated that the presence of natural elements in noise-exposed sites have a moderating influence on people’s noise responses. Links between landscape and health have long been observed in many different cultures and societies. Langdon found that high neighborhood quality was associated with attractive appearances, whereas the presence of parks and green spaces lowered dissatisfaction with traffic noise significantly in a large survey of nearly 3,000 people in 53 residential sites of London [4]. Urban parks and open green spaces are of strategic importance for reducing stress, promoting health and well-being [5], enhancing contemplativeness and providing a sense of peacefulness and tranquility [6]. In recent years, many researchers have highlighted the role of environmental psychology in environmental evaluation. During the development of the field of environment psychology, several environmental therapeutic theories have been put forward by Ulrich [7,8] and by Kaplan and Gesler [9,10]. These theories describe the restorative effect of natural elements such as trees, grass, bushes and lakes on human physical and psychological fatigue [9,10]. Viewing natural landscapes (e.g., vegetation, water and other natural elements) generally creates a stronger positive health effect than viewing urban landscapes (e.g., concrete, buildings, and other man-made structures), a claim supported by Ulrich’s “Stress Recovery Theory” (SRT) [7,8] and the “Attention Restoration Theory” (ART) of Kaplan and Kaplan [9]. Experiments by Tennessen & Cimprich and Berto have provided support for the ART theory that restorative environments help maintain and restore the capacity to direct attention [11,12].

Other studies have investigated the relationships between acoustics and vision in urban environments and their effects on health and well-being. For example, natural and silent visual images (e.g., fewer buildings and plenty of open spaces) can increase preference for an environment with noisy transportation and human activities and can enhance a sensation of inactiveness and silence because of the gap between the visual and auditory stimuli [13]. The potential for green-area availability to moderate residents’ responses to noise is an important effect that has been demonstrated by Gidlöf-Gunnarsson and Öhrström [14]. They found that having access to a quiet area nearby one’s dwelling, even in areas exposed to heavy noise pollution, lessened noise annoyance and improved many basic health qualities including stress-related psychosocial symptoms and sleep. In summary, previous studies have demonstrated that natural and semi-natural environments and urban green spaces can affect people’s emotions, and they emphasize the specific influences of audio and visual elements obtained from the natural environment.

Our study focuses on the psychological effects (psychological noise reduction) of visual sensations from the nature environment and how psychological noise reduction by means of landscaping can achieve improvements in health benefits and psychological behavior (e.g., help with recovery from stress and/or mental fatigue and eliminate fidgeting).

## 2. Method

### 2.1. Equipment

We took videos of noisy street scenes and adjacent green spaces using a digital videocon (SONY DCR-PC300K). The environmental noise was measured using a sound level meter (AWA6128B, Aihua Co. Ltd., Hangzhou, China). The noise was simulated using an AWA6290A multi-channel noise and vibration analyzer (Aihua Co., Ltd., Hangzhou, China), and it was played with a KMS-EV1010 (KMS Co., Ltd., Guangzhou, China) loudspeaker as a noise source. The video was played with Itheater-VG920C video glasses (Itheater Co. Ltd., Shanghai, China). The electroencephalogram (EEG) was detected and recorded using an SP-Mars II portable electroencephalograph (Siga Medical Equipment Co., Ltd., Shanghai, China), which has 16 EEG register channels (C3, C4, F3, F4, F7, F8, Fp1, Fp2, O1, O2, P3, P4, T3, T4, T5 and T6).

### 2.2. Experimental Design

**Figure 1.**
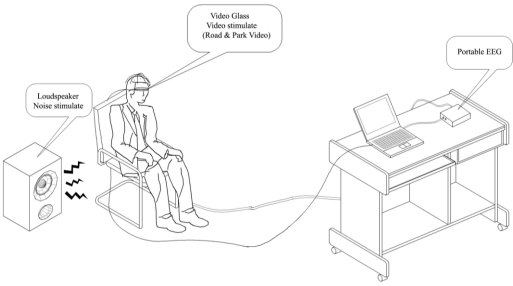
Diagram of the experimental design.

As mentioned above, the experimental stimuli emphasized the visual aspect of the experience and its psychological effects. If the surveys were taken outdoors (such as in urban parks or natural reserves), then they would be unavoidably influenced by the vegetation’s physical effect on noise reduction. In addition, several studies show that health benefits related to experiencing Nature have been based on opportunities for noticing and observing Nature, rather than on performing activities in Nature [15]. Therefore, the experiment was conducted in a laboratory with the same noise volume and recorded visual stimuli to ensure that the background was uniform for all of the subjects and that the results were therefore precise. Previous studies have constructed landscapes using photographs, slide shows or other still images. However, these images are typically far from realistic. In our case, we began with the idea that the method used in a project such as this should not only prove controllable and uniform for survey participants, but it should also be sufficiently effective at expressing and representing the actual environment so as to overcome the above-mentioned difficulties. This can be achieved through the use of semi-actual stimuli: videos played through video glasses and recorded sounds (Figure 1). The evaluation methods of the previous investigations were mainly qualitative and subjective, including observations, self-reports, questionnaires and structured interviews. In this experiment, the electroencephalogram (EEG) was chosen to obtain quantitative emotional responses in addition to the qualitative evaluation of questionnaires.

**Figure 2.**
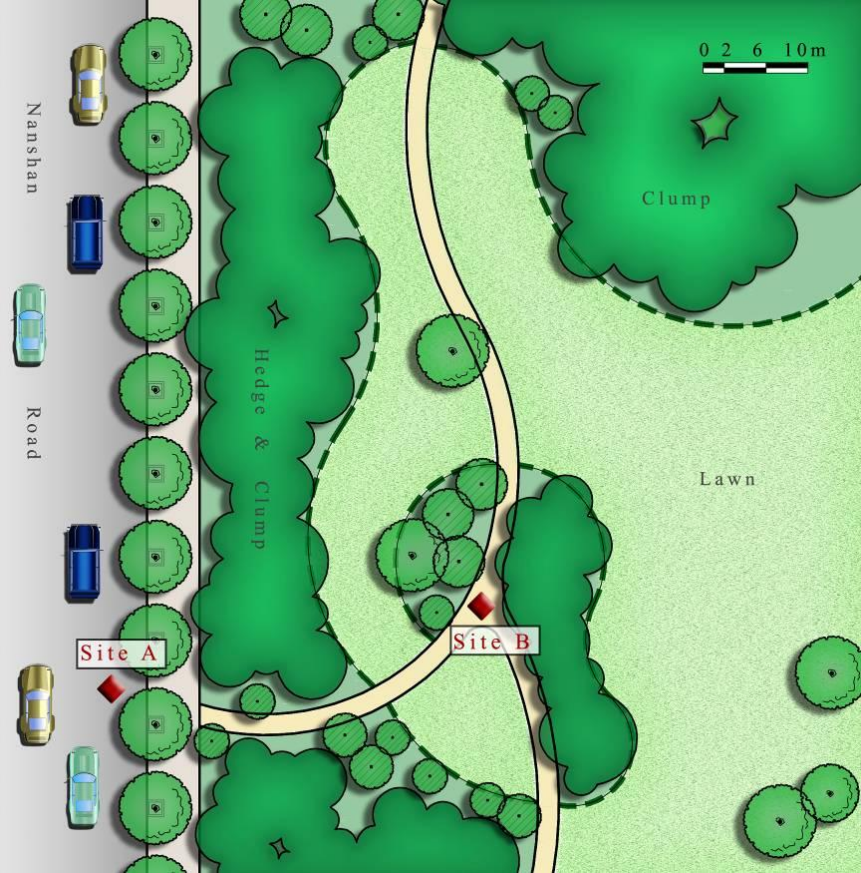
Diagram of the shooting scene.

Before the survey, we took videos of a busy road (Nanshan Road, Hangzhou) and the vegetation next to the road. The traffic flow down this road was approximately 808 vehicles per hour (8:00 AM ∼12:00 AM, data from the Hangzhou Traffic Management Bureau; Figure 2). We recorded traffic scene samples at Site A and vegetation scene samples at Site B. Both of the video samples were edited into a three minute clip. The noise level (L_Aeq_) of Site A was 68.6 dB, and that at Site B was approximately 62.9 dB as averaged over three surveys per day with the sound level meter. These tasks were performed as preparations for the lab experiment.

The lab experiment began with participants completing a questionnaire; they had previously been briefed about the experiment’s aims, content and safety. The questionnaire consisted of three parts: (1) the background of the responder; (2) the responder’s evaluation of the acoustical condition of their living environment; and (3) the responder’s attitude toward the idea that landscape plants can reduce noise. In the questionnaire, multiple-choice items and semantic profiles were the primary means of asking questions. The experiment was then performed in three steps, as follows:

1. The electroencephalogram (EEG) value (P_1_) of the responder was recorded three minutes after they wore the video glasses and portable electroencephalograph to avoid impacts of unfamiliar equipment on the responder’s EEG. No video on the glasses or noise from the loudspeaker was used as the BC (black controller) set.
2. The electroencephalogram (EEG) value (P_2_) of the responder was recorded with the video of Site A (road traffic and passers-by, an image of people walking on the sidewalk) playing on the glasses and with the noise played from the loudspeaker. The volume was regulated to ensure that the L_Aeq_ value of the lab was 68.6 dB. This step was also recorded over a three minute period.
3. The electroencephalogram (EEG) value (P_3_) of the responder was recorded with a video of Site B (hedges, lawns and other vegetation forms in street parks, an image of people walking in the park next to the road) playing on the glasses and noise produced from the loudspeaker. The L_Aeq_ value was 68.6 dB. This step was also recorded over a three minute period.

**Figure 3.**
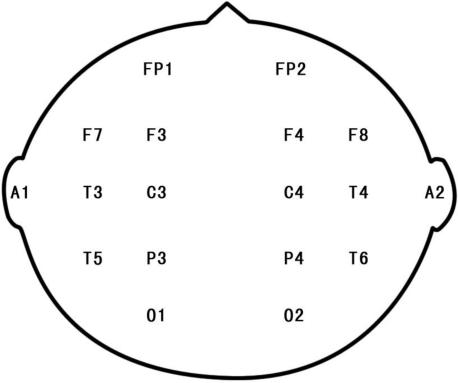
Diagram showing the locations of the EEG electrodes (the figure shows the head of a subject).

All the noises broadcast during the experiment were the same, as translated by the AWA6290A multi-channel noise and vibration analyzer. There was a two minute break between each of the three steps to provide a break and to lessen the effects of fidgeting and emotions accumulated during the previous step. The experimental subjects were seated in a dim and noiseless room. The 16-channel electrodes were located according to the ISO10-20 system. Reference electrodes were placed on the left and right ear (A1 + A2). EEG electrodes were collapsed into 16 clusters. This procedure resulted in eight regional means for each hemisphere: frontopolar—Fp (Fp1, Fp2); frontal—F (F3, F4, F7 and F8), central—C (C3, C4); temporal—T (T3, T4, T5 and T6); parietal—P (P3, P4) and occipital—O (O1, O2; Figure 3). Data were recorded at sampling rates of 256 Hz and 12 bits using an A/D converter. The EEG was grouped into the delta (0.1–3.5 Hz), theta (4–7.5 Hz), alpha-1 (8–11.0 Hz), alpha-2 (11.5–13.5 Hz), beta-1 (14–18.5 Hz) and beta-2 (19–30 Hz) frequency bands.

### 2.3. Participants

A total of 40 survey participants (20 female and 20 male), students from Zhejiang Forestry University aged 21 to 25 years (with an average age of 23 years), were used in this study. Participants were randomly selected within the university and were given a simple oral introduction to the survey methods before being invited to participate in the study.

## 3. Results

### 3.1. The Subjective Emotional Evaluation

The questionnaire results showed that 75% of the responders thought that the noise in their living environment was indifferent and tolerable, whereas 17.5% of them were disturbed by the noise and could not stand it, and the remaining 7.5% thought that the noise was disturbing and annoyed them at times. The highest satisfaction rates among the subjects were reported by students living on campus. The university thus appears to be much more quiet and peaceful than other urban environments. Nevertheless, all of the subjects considered noise to be the foremost environmental problem, being more disruptive than other disturbances such as air pollution, solid waste and water pollution. The subjective initial response to noise pollution was investigated in the questionnaire through the following question: how do you deal with unpleasant noise when it arises? Of the subjects, 77.5% responded that they would ‘leave the noise source as soon as possible and find another quiet and comfortable environment’ (Answer A); 12.5% of respondents indicated that they would suffer silently and hope that the noise would be reduced or would fade away (Answer C); 7.5% of respondents indicated that they would stop the source from producing any more noise (Answer B); and the remaining 2.5% responded that they would make noise to disturb others in order to force someone else to stand up against the unpleasant noise (Answer D).

The questionnaire results imply that the majority of people are negatively affected by noise and have a passive attitude toward shielding the noise. According to these results, providing an oasis of serenity amid chaos is the best way to release urban residents’ stress and calm their nerves.

### 3.2. Perceived Noise Reduction Provided by Landscape Plants

**Figure 4.**
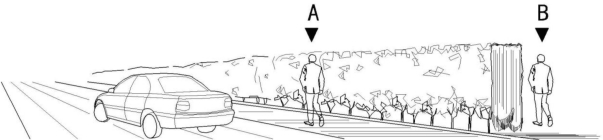
Diagram illustrating sites A and B described in the questionnaire.

Through the questionnaire survey, we found that 90% of the subjects believed that landscape plants could contribute to noise reduction, 7.5% were unsure and 2.5% disagreed. As for the prominent belief in the noise reduction provided by landscape plants, 80% of the participants indicated that plant hedges were the most effective noise barriers. Concrete and plastic noise barriers each had 10% supporters whereas metal barriers were not chosen as the most effective barrier by any of the survey respondents. To quantify the ability of plants to function as a barrier to attenuate noise, the subjects were asked to estimate the noise decibel gap between sites A and B, as shown in Figure 4 through five given answers.

The five options that respondents could select from were as follows: >10 dB, 8–10 dB, 5–8 dB, 3–5 dB and <3 dB. Of the respondents, 30% and 25% thought that the noise decibel gap between the sites would be more than 10 dB and 8–10 dB, respectively. They overrated the plants’ ability to attenuate noise. Forty percent of respondents chose a noise decibel gap of 5–8 dB, which is the approximate value of the actual noise attenuation and the remaining 5% chose 3–5 dB, underrating the noise attenuation provided by the hedge. Based on this part of the questionnaire, we conclude that the landscape plants were thought to be highly effective noise barriers and that their effectiveness was even overrated. In other words, the overrated noise attenuation caused by a person’s subjectivity can be described as a psychological noise reduction. Different types of mental activities, ranging from visual processing to negative emotions, are associated with distinct types of brain activity relative to rest conditions. We used EEGs to investigate the objective existence of psychological noise reduction.

### 3.3. EEG Values

Mean values of the EEG power during the three conditions are shown in Table 1 for all frequency bands. The values exported by the SP-Mars II portable electroencephalograph were the mean square of EEG power voltage, so the unit for these values should be μV^2^. Both beta-1 and beta-2 power at F3, F4, F7, F8, Fp1, Fp2, O1 and O2 increased significantly more in the traffic scene group than in the vegetation scene group or BC group (P < 0.05). Neither the alpha-1 nor alpha-2 band differed significantly among the three groups at any of the recording sites, except for the alpha-2 band at the Fp2 site. At this site, both the traffic scene group and the vegetation scene group decreased significantly more than did the BC group (P < 0.05). There were no significant differences in the theta band between the traffic scene and the vegetation scene groups at any electrodes, and the vegetation scene and BC groups also failed to reach significance. However, there was a significant difference in values between the traffic scene and BC groups except for the central sites (C3, C4). This finding partially agrees with earlier reports of strong theta band increases during concentrated task performance [16] and memory operations [17]. The delta band was most like the theta band in that the differences between the sets were not significant and were much more intricate. Within electrophysiological studies of emotion, there is no tangible correlation between the delta band and emotional processing, so we have not investigated the delta band further in the following text.

Findings from EEG studies show that the relative per centum of the six frequency bands may help to elucidate emotional processing [18]. In the present study, the relative per centum of every band is given by:

(1) $$\left[\text{P}={\text{P}}_{2,\;3}-{\text{P}}_{1}\right]$$

where P_2_ and P_3_ are the relative per centum of the EEG recorded during the traffic and plant scenes, and P_1_ is the value of the BC set. The results of a single-factor ANOVA performed on these data are shown in Table 2. The delta and theta bands were not significant at any electrode. There was significantly less alpha activity in the vegetation scene than in the traffic scene at frontopolar, central, parietal and occipital regions. This finding supports prior research showing some decreased alpha activity involvement in emotional processing [18]. Furthermore, beta activity increased significantly more with the traffic scene than with the plant scene at all sites except T3, T4.

Interestingly, the present data suggest that the right hemisphere was more emotionally active than the left for all frequency bands because both the EEG power (Table 1) and the percent fluctuation (Table 2) of the even electrodes located on the right hemisphere were larger than those of the odd electrodes located on the left hemisphere. As shown in other studies, greater right hemisphere activity may be associated with elevated negative emotions, withdrawal and/or anxiety [18–21].

## 4. Discussion

### 4.1. Interaction between Auditory and Visual Element

Through a literature review, we found that the landscape stimuli used in studies comparing the health outcomes of different landscapes were generally simple, were mainly focused on vision, and the category comparisons were generally very coarse, primarily using two categories: exposure to natural *versus* urban landscape views and landscape views *versus* no views. Though vision is by far our most important sense in terms of yielding information about outdoor environments [22], environmental perception is clearly multi-sensory and is not restricted to vision. Accordingly, sound is becoming an increasingly important research subject within the field of urban environmental science.

As the concept of a soundscape becomes established, several researchers have become increasingly concerned with the interaction of visual and auditory elements in urban environments. Tamura assessed the capacity of various landscapes to induce feelings of annoyance [23]. The results indicated that the feeling of annoyance was a combination of both auditory and visual factors. Viollon, Lavandier, and Drake examined the influence of visual settings on sound ratings in an urban environment, and again the results showed a significant and multi-faceted visual influence [24]. Ge and Hokao concluded that in areas with natural visual imagery but noisy transportation and human activities, visual information can change the perception of the soundscape a great deal [13]. As a contrast to the traffic-dominated environment, green landscape plants are highly complex with respect to content and structure (*i.e.*, the “extent” component of ART), and such environments require less directed attention from subjects and allow them to rest and feel restored [9,12,25,26]. If green areas are perceived as visually attractive, they may also help to reduce stress (e.g., due to traffic noise) by creating pleasant and calm feelings [5,27]. In our survey, the EEG results showed that there is a significant difference in human physiological responses to vegetation and traffic views. The EEG results also indicated that landscapes such as vegetation, water and other natural elements have positive effects on physiological health and psychological well-being regardless of whether urban sounds accompany the visual observations. In other words, visual stimuli partially influence the psychological apperception of acoustic perceptions.

### 4.2. Psychological Responses to Environmental Stimuli

Psychological responses to environmental stimuli are linked with various mediating and/or moderating factors that relate to the individual (e.g., noise sensitivity, coping style) and the environment (e.g., predictability and control of the noise). The environment has the potential to influence the psychological process and the impact of stimuli on psychological responses, and we therefore controlled the simulation of environment stimuli and the quantitative EEG scaling means (which can be objective) to ensure that the survey was accurate. The validity of the different landscape stimuli was a key factor in the evaluation of the restorative environments. A comprehensive review indicated that restorative environment studies have been conducted primarily by using images of landscapes (such as from a window, or a photograph, *etc.*), and the remaining studies have been based on activities in real landscapes wherein the treatments differed with respect to the landscape type in the area where the activities were performed [28]. Hartig, Böök, Garvill, Olsson and Gärling compared evaluations of restorative quality obtained by on-site visits with those from simulations and found no statistically significant difference between the two treatments [29]. This finding suggests that simulations are likely to be a valid means of evaluating the restorative potential of a landscape. Furthermore, it has been demonstrated that viewing natural settings can produce significant restoration within five minutes, as indicated by positive changes in physiological parameters (e.g., blood pressure, heart rate, and muscle tension) [30]. The means of simulating environmental stimuli in this paper were derived from the literature with the goal of improving the representation of actual environmental stimuli.

The observed EEG values indicate that vegetation reduced psychological stress markedly, providing an impressive example of the restorative effects of green spaces on the psychological and physiological processes of human beings. Compared with the presence of road traffic and urban structures in the visual field, the presence of vegetation and other green areas are linked to a higher alpha percentage and a lower beta percentage, which indicates the presence of a positively perceived emotional difference [18]. Thus, psychological responses are significantly linked with environment stimuli, especially annoyances caused by road traffic noise, as found in previous studies [31,32]. Langdon and Lercher investigated the influence of natural elements on noise reaction and suggested that there was a link between more attractive visual appearances in the noisy environment and higher perceived neighborhood quality [4,32]. Johansson pointed out that the presence of vegetation positively affects the perceptions of a ventilation noise-contaminated environment [33], and this has been demonstrated by several other researchers [24,34,35]. Our study demonstrates the moderating effect of visible vegetation on noise responses, which is in agreement with the findings of the previous studies mentioned above.

### 4.3. Congruence of the EEG Evaluations with the Questionnaire Results

A highly significant asymmetry has been observed between the EEG activity of the vegetation scene group and that of the traffic scene group, and the EGG activity of the right hemisphere is more important than that of the left hemisphere because more right-sided parietal EEG activity in the beta frequency domain has been found to be indicative of a more avoidant response to angry facial expressions [18]. Focused psycho-physiological studies with sophisticated simulated scenarios are needed to reliably determine the neurobehavioral concomitants of these two motivational systems and their roles in human anxiety [20]. Both the quantitative (EEG evaluation) and qualitative (the questionnaire survey) methods used in this experiment indicate that landscape plants can cause exaggerated levels of noise reduction due to expressed and self-suggested psychological noise reduction. The EEG data indicate that the subjects’ frame of mind is significantly calmer in vegetation scenes than in traffic scenes, even when the subjects are exposed to the same traffic noise in the two scenes. The questionnaire also revealed that 55% of the subjects overrated the plants’ ability to attenuate noise, which provides further evidence of subjective psychological noise reduction. These results corroborate previous observations regarding the psychological noise reduction capabilities of plants [5,14,22,36,37]. Thus, both the objective (quantitative) and subjective (qualitative) methodologies employed herein indicate that plants can induce psychological noise reduction.

## 5. Conclusions

Three important points should be emphasized in this study. First, landscape plants provide excess noise attenuation to subjects’ emotional processing, a phenomenon termed psychological noise reduction in this paper. The green environment of slow transport systems, which primarily uses landscape plants and is largely concerned with roadside green space, aims to moderate the tension caused by traffic noise and is concerned with increasing the probability of walking trips so as to improve the popularity of ‘Green Transportation’. In the present study, psychological noise reduction was confirmed both by a questionnaire survey, which provided subjective evidence of mental activity, and by EEG data, which provided objective evidence of physiological processes pertinent to psychology. This finding is consistent with the environmental psychology literature, which is concerned with the influence of landscape on health [9,12,14,25,26,28]. Second, the findings from this study suggest that the emotional activity aroused by noise and visual stimuli is manifested in the synchronization of beta frequency bands and the desynchronization of alpha frequency bands. These observations are in agreement with the findings of previous EEG environmental psychology studies [5,17,36]. Finally, the EEG patterns induced by these two emotional stimuli indicate that they activate different regions of the brain. In other words, the frontopolar, central, parietal and occipital regions are much more sensitive to emotion aroused by noise. Furthermore, the right hemisphere has been found to be more emotionally active than the left during negative emotional conditions, which agrees with Luo, Tang and Xiong [18]. The evaluation and assessment of these intangible services and benefits are of crucial importance to justifying and legitimizing strategies for urban sustainability.

It is important to note a limitation of our study. We studied only students at Zhejiang Forestry University, which constitutes a biased sample of subjects. The citizens that suffer the most from noisy urban environments are those that are situated outside of the university and range from children to the elderly, particularly those who enjoy recreational activities in street parks and those living close to main roads. These individuals are mainly middle aged and elderly. As a result, the potential for individual differences in physiological function due to age cannot be neglected. Replication is needed to address these potential age differences. Because this study provided evidence for psychological noise reduction, we plan to conduct further investigations into how the landscape impacts emotional processes in a range of individuals.

## Figures and Tables

**Table 1.** The EEG power (μV^2^) result.

**Frequency bands**	**Set**	**EEG power (μV^2^)**			
		**C3**	**C4**	**F3**	**F4**

δ	1	663.02(220.08)a	688.15(220.73)a	800.05(205.38)b	830.40(192.89)b
	2	750.37(254.12)a	771.47(255.83)a	920.83(235.29)a	942.98(236.42)a
	3	680.70(236.45)a	705.98(245.99)a	820.49(208.14)b	842.60(192.91)b
θ	1	581.42(247.12)a	601.17(253.74)a	662.81(257.88)b	684.23(267.23)b
	2	700.80(300.27)a	714.35(295.08)a	798.76(297.52)a	835.36(308.17)a
	3	630.01(274.30)a	645.11(269.18)a	727.19(282.61)ab	746.89(273.59)ab
α_1_	1	413.08(171.27)a	434.72(151.11)a	435.27(171.07)a	449.61(154.38)a
	2	456.21(186.61)a	473.05(173.41)a	483.42(188.07)a	498.38(175.30)a
	3	422.20(181.39)a	438.50(160.79)a	450.91(170.94)a	462.13(150.86)a
α_2_	1	237.53(103.88)a	260.62(112.71)a	231.96(82.69)a	237.38(78.64)a
	2	232.24(117.44)a	246.30(120.21)a	201.47(100.81)a	200.46(96.64)a
	3	222.73(105.51)a	237.25(114.83)a	194.59(89.38)a	199.91(85.74)a
β_1_	1	352.58(132.10)b	359.58(127.74)b	**355.71(98.30)c**	**367.74(94.42)c**
	2	453.93(157.99)a	464.57(149.63)a	**490.43(153.71)a**	**521.06(139.78)a**
	3	398.43(143.18)ab	408.64(138.21)ab	**418.78(109.26)b**	**438.54(107.05)b**
β_2_	1	675.71(195.27)b	724.34(196.71)b	**770.38(221.08)c**	**766.18(218.91)c**
	2	868.52(241.38)a	923.71(226.46)a	**1046.28(309.48)a**	**1047.54(288.37)a**
	3	764.01(208.62)b	816.64(203.80)b	**922.31(253.18)b**	**920.09(243.49)b**

**Frequency bands**	**Set**	**EEG power (μV^2^)**			
		**F7**	**F8**	**FP1**	**FP2**

δ	1	901.88(199.66)b	921.76(197.17)b	969.15(248.08)b	994.83(237.92)b
	2	1058.57(219.09)a	1071.26(209.11)a	1183.88(276.51)a	1207.95(272.93)a
	3	971.62(195.64)ab	974.64(181.28)b	1071.89(258.19)ab	1088.09(249.26)b
θ	1	767.37(215.12)b	797.59(231.28)b	807.73(278.10)b	826.38(267.76)b
	2	906.02(249.61)a	928.07(240.43)a	1010.59(283.59)a	1032.88(275.73)a
	3	821.31(213.97)ab	842.24(217.30)ab	907.70(275.22)ab	935.13(268.47)ab
α_1_	1	498.57(148.04)a	537.73(156.04)a	503.42(157.98)a	520.98(152.96)a
	2	540.04(173.45)a	570.42(173.44)a	550.90(178.04)a	540.07(159.10)a
	3	498.11(134.94)a	524.86(145.11)a	505.15(153.36)a	509.94(147.57)a
α_2_	1	285.42(82.23)a	307.49(95.99)a	272.97(99.69)a	282.31(100.41)a
	2	286.90(104.82)a	291.96(114.19)a	250.40(118.44)a	218.78(115.81)b
	3	273.45(90.57)a	278.70(96.98)a	234.99(108.72)a	222.94(107.56)b
β_1_	1	**428.01(112.21)c**	**469.53(119.05)c**	**461.89(160.94)c**	**493.39(164.81)c**
	2	**567.37(152.41)a**	**618.18(155.15)a**	**678.49(219.15)a**	**750.76(226.17)a**
	3	**494.15(117.89)b**	**541.16(123.98)b**	**574.54(196.03)b**	**640.15(212.50)b**
β_2_	1	**921.40(230.14)c**	**936.86(233.54)c**	**937.40(289.10)c**	**986.05(287.50)c**
	2	**1187.38(288.23)a**	**1196.43(287.65)a**	**1285.29(317.99)a**	**1381.34(309.28)a**
	3	**1044.67(243.36)b**	**1057.85(242.25)b**	**1123.49(295.09)b**	**1203.56(301.50)b**

**Frequency bands**	**Set**	**EEG power (μV^2^)**			
		**O1**	**O2**	**P3**	**P4**

δ	1	927.06(139.30)a	965.93(139.47)b	702.97(160.40)b	768.92(168.01)b
	2	**1030.**90(155.38)a	1075.07(157.83)a	846.99(209.09)a	904.63(221.57)a
	3	958.81(141.17)b	999.61(139.99)b	745.37(188.84)b	795.93(206.37)b
θ	1	829.68(189.25)b	847.42(170.74)b	653.27(255.62)b	692.94(252.62)b
	2	953.90(219.53)a	974.45(196.79)a	792.00(297.00)a	820.18(285.87)a
	3	875.80(195.00)ab	895.19(172.99)ab	705.87(269.31)ab	736.30(275.41)ab
α_1_	1	610.81(199.94)a	635.23(211.01)a	513.85(228.24)a	554.75(233.13)a
	2	624.32(224.26)a	637.78(233.18)a	564.05(272.10)a	577.72(276.15)a
	3	587.14(196.63)a	603.21(210.49)a	505.74(231.09)a	530.07(228.79)a
α_2_	1	378.61(150.61)a	401.39(146.28)a	298.77(136.37)a	322.71(142.05)a
	2	332.53(167.37)a	344.21(163.63)a	289.90(140.51)a	292.82(137.37)a
	3	329.25(151.34)a	339.81(146.07)a	275.00(122.86)a	281.47(125.62)a
β_1_	1	**575.05(143.94)c**	**598.16(147.14)c**	404.03(152.84)b	447.14(159.72)b
	2	**741.61(183.86)a**	**781.18(184.89)a**	550.36(187.86)a	601.18(191.19)a
	3	**665.27(164.53)b**	**704.51(168.33)b**	467.84(160.13)b	517.60(170.49)b
β_2_	1	**1030.17(179.50)c**	**1087.75(206.16)c**	788.86(202.63)b	828.93(194.03)b
	2	**1283.36(240.92)a**	**1357.82(251.5)a**	1019.76(256.81)a	1056.20(249.38)a
	3	**1161.15(207.75)b**	**1233.25(229.13)b**	886.41(227.93)b	923.84(216.33)b

**Frequency bands**	**Set**	**EEG power (μV^2^)**			
		**T3**	**T4**	**T5**	**T6**

δ	1	859.41(217.81)a	903.72(215.80)a	855.60(164.84)b	891.04(153.47)b
	2	961.51(281.16)a	989.06(278.12)a	989.40(172.93)a	995.70(164.19)a
	3	875.85(250.45)a	900.54(255.16)a	873.26(174.09)b	908.19(166.68)b
θ	1	712.92(228.49)b	755.00(278.29)b	744.44(217.05)b	786.19(205.01)b
	2	866.18(294.80)a	899.50(320.81)a	889.79(255.60)a	929.04(243.47)a
	3	780.30(246.70)ab	813.58(281.50)ab	840.89(231.96)ab	848.94(209.41)ab
α_1_	1	510.64(173.55)a	538.78(163.32)a	553.36(211.63)a	598.54(231.66)a
	2	556.34(200.39)a	566.75(181.01)a	607.77(228.41)a	639.01(251.49)a
	3	500.86(168.60)a	518.37(154.74)a	554.00(192.90)a	581.90(218.04)a
α_2_	1	291.08(96.21)a	312.07(104.02)a	347.88(127.95)a	378.57(151.40)a
	2	296.92(108.99)a	305.46(120.02)a	366.57(155.32)a	374.40(180.01)a
	3	271.81(99.47)a	289.85(110.00)a	329.63(130.88)a	343.98(146.83)a
β_1_	1	**423.12(105.40)c**	**472.11(117.43)c**	483.59(135.31)b	523.09(134.18)b
	2	**571.17(155.94)a**	**627.49(162.79)a**	626.51(197.29)a	676.26(196.15)a
	3	**507.73(143.28)b**	**557.94(147.34)b**	568.77(152.68)a	608.76(154.09)a
β_2_	1	897.70(295.07)b	948.24(288.22)b	**919.44(210.66)c**	**947.20(214.65)c**
	2	1131.44(340.89)a	1182.56(321.27)a	**1187.28(304.22)a**	**1206.66(288.48)a**
	3	1010.01(325.23)ab	1058.96(305.56)ab	**1058.64(244.87)b**	**1071.10(248.34)b**

The data presented are means with SD in parentheses; differences are significant at P < 0.05 according to a Tukey’s test. Set 1: the BC set; Set 2: the Site A (traffic) scene set; Set 3: the Site B (landscape plant) scene set.

**Table 2.** Results of an ANOVA for the relative per centum of the six frequency EEG bands (data are presented as means with SD in parentheses, * P < 0.05, ** P < 0.01).

**Frequency bands**	**Set**	**Site**			
		**C3**	**C4**	**F3**	**F4**

δ	traffic scene	−1.09(1.19)	−1.14(1.19)	−1.28(1.05)	−1.63(1.00)
	plant scene	−0.93(1.10)	−0.90(1.05)	−1.49(1.28)	−1.69(1.23)
θ	traffic scene	0.34(0.85)	0.37(0.87)	0(0.73)	0.18(0.61)
	plant scene	0.25(0.67)	0.30(0.69)	0.21(0.74)	0.26(0.81)
α1	traffic scene	−0.93(0.34) **	−1.06(0.36) **	−0.97(0.39) **	−1.11(0.52) **
	plant scene	−0.60(0.26) **	−0.71(0.25) **	−0.49(0.40) **	−0.59(0.47) **
α2	traffic scene	−1.40(0.56) **	−1.61(0.45) **	−2.03(0.54) **	−2.20(0.47) **
	plant scene	−0.97(0.30) **	−1.21(0.36) **	−1.61(0.50) **	−1.60(0.57) **
β1	traffic scene	1.07(0.44) **	1.25(0.48) **	1.42(0.65) **	1.80(0.52) *
	plant scene	0.76(0.33) **	0.87(0.36) **	0.93(0.59) **	1.10(0.51) *
β2	traffic scene	2.01(0.64) **	2.20(0.62) **	2.85(0.53) **	2.95(0.54) *
	plant scene	1.49(0.65) **	1.65(0.63) **	2.45(0.73) **	2.51(0.72) *

**Frequency bands**	**Set**	**Site**			
		**F7**	**F8**	**FP1**	**FP2**

δ	traffic scene	−0.34(1.42)	−0.21(1.38)	−0.8(1.62)	−0.83(1.38)
	plant scene	0.02(1.00)	−0.04(1.20)	−0.42(1.62)	−0.63(1.37)
θ	traffic scene	−0.29(0.78)	−0.21(0.82)	0.07(1.02)	0.09(0.67)
	plant scene	−0.20(0.69)	−0.14(0.79)	0.18(1.10)	0.23(0.83)
α1	traffic scene	−1.28(0.53) **	−1.36(0.56) *	−1.66(0.58) **	−2.18(0.61) **
	plant scene	−0.94(0.57) **	−1.09(0.52) *	−1.28(0.53) **	−1.60(0.57) **
α2	traffic scene	−1.22(0.55) **	−1.58(0.53) **	−1.93(0.55) *	−2.67(0.59) **
	plant scene	−0.91(0.43) **	−1.19(0.43) **	−1.62(0.52) *	−2.07(0.62) **
β1	traffic scene	1.20(0.40) **	1.37(0.46) **	1.97(0.38) **	2.55(0.49) **
	plant scene	0.79(0.35) **	0.99(0.43) **	1.29(0.36) **	1.82(0.49) **
β2	traffic scene	1.92(0.58) **	2.00(0.51) **	2.35(0.54) **	3.03(0.48) **
	plant scene	1.24(0.57) **	1.46(0.48) **	1.86(0.54) **	2.24(0.56) **

**Frequency bands**	**Set**	**Site**			
		**O1**	**O2**	**P3**	**P4**

δ	traffic scene	−0.36(1.31)	−0.37(1.27)	−0.05(1.60)	−0.03(1.62)
	plant scene	−0.27(1.13)	−0.27(1.16)	−0.21(1.35)	−0.35(1.32)
θ	traffic scene	0.10(0.76)	0.13(0.78)	0.08(0.72)	0.14(0.75)
	plant scene	0.04(0.68)	0.05(0.74)	0.19(0.75)	0.19(0.73)
α1	traffic scene	−1.51(0.56) **	−1.69(0.51) **	−1.47(0.60) **	−1.82(0.66) **
	plant scene	−1.18(0.53) **	−1.37(0.54) **	−1.12(0.45) **	−1.25(0.51) **
α2	traffic scene	−2.04(0.45) **	−2.24(0.49) **	−1.76(0.59) **	−2.01(0.58) **
	plant scene	−1.52(0.55) **	−1.75(0.53) **	−1.16(0.52) **	−1.45(0.57) **
β1	traffic scene	1.68(0.36) **	1.87(0.38) **	1.59(0.49) **	1.82(0.47) **
	plant scene	1.28(0.41) **	1.51(0.41) **	1.09(0.53) **	1.35(0.50) **
β2	traffic scene	2.14(0.39) **	2.31(0.43) **	1.62(0.50) **	1.90(0.45) **
	plant scene	1.65(0.39) **	1.84(0.44) **	1.21(0.50) **	1.50(0.47) **

**Frequency bands**	**Set**	**Site**			
		**T3**	**T4**	**T5**	**T6**

δ	traffic scene	−2.03(1.27)	−2.25(1.38)	−1.43(1.57)	−1.62(1.85)
	plant scene	−1.91(0.92)	−2.16(1.08)	−1.26(1.51)	−1.13(1.58)
θ	traffic scene	1.03(0.77)	1.36(1.04)	0.85(1.06)	1.09(1.23)
	plant scene	0.73(0.68)	1.16(0.86)	0.50(0.84)	0.71(0.87)
α1	traffic scene	−1.12(0.45)	−1.36(0.46)	−0.95(0.53)	−1.24(0.54)
	plant scene	−0.96(0.49)	−1.18(0.43)	−0.77(0.65)	−1.11(0.63)
α2	traffic scene	−1.10(0.43)	−1.33(0.44)	−1.09(0.49)	−1.48(0.58)
	plant scene	−1.02(0.44)	−1.18(0.41)	−0.92(0.54)	−1.30(0.55)
β1	traffic scene	1.55(0.49)	1.70(0.44)	0.95(0.64)	1.25(0.52)
	plant scene	1.52(0.50)	1.63(0.45)	1.09(0.55)	1.26(0.42)
β2	traffic scene	1.68(0.41)	1.89(0.39)	1.67(0.60) **	2.00(0.46) **
	plant scene	1.65(0.46)	1.73(0.39)	1.36(0.57) **	1.58(0.44) **
